# Spatial distribution characteristics and influencing factors of tourism resources based on point of interest data

**DOI:** 10.1371/journal.pone.0310487

**Published:** 2024-09-18

**Authors:** Feng Ding, Shijun Zhang, Luote Dai

**Affiliations:** 1 Department of Business and Trade, Zhejiang Industry Polytechnic College, Shaoxing, Zhejiang, China; 2 Digital Economy and Trade College, Wenzhou Polytechnic, Wenzhou, Zhejiang, China; University of Peshawar National Centre of Excellence in Geology, PAKISTAN

## Abstract

The agglomeration and dispersion of tourist attractions in space greatly affect the development of regional tourism resources and the consumption choice of tourism market. At present, the research on the spatial distribution characteristics of tourist attractions and their influencing factors mainly adopts induction and investigation, and there is a lack of effective statistical models for the research on the spatial distribution of tourist attractions and their influencing factors in some historical and cultural ancient cities. This paper uses Internet technology to obtain the spatial distribution data of tourist attractions in Shaoxing city, and uses mean nearest neighbor analysis, nuclear density analysis, imbalance index analysis, standard deviation ellipse and other spatial statistical analysis techniques and geographical detector methods to study the spatial distribution characteristics and influencing factors of tourist attractions in Shaoxing City. This paper studied the distribution characteristics of tourist attractions in Shaoxing city, such as spatial aggregation, distribution equilibrium and spatial orientation, and applied geographical detector to study the influencing factors of the spatial distribution of scenic spots. It was concluded that the spatial distribution pattern of scenic spots was affected by various factors such as natural environment, social environment and economic environment. The explanatory power of two-factor interaction is obviously stronger than that of single factor. The research results provide scientific basis for the planning, layout and development of tourist attractions in Shaoxing and its similar cities, and then promote the high-quality development of tourism in Shaoxing and its similar historical and cultural ancient cities.

## Introduction

At present, tourism has become an important driving force for the sustainable development of China’s regional economy [1], and the spatial distribution characteristics of tourism resources to a large extent affect the development of regional tourism resources and consumption choices in the tourism market, and then affect the overall development of tourism [2]. The Zhejiang provincial government has clearly proposed to optimize the spatial layout of tourism development.

In the process of social and economic development, there will inevitably be connections among neighboring economic objects, thus forming aggregation. The aggregation area can be optimized to form the best spatial structure and achieve the best development [3]. For example, Xue Xiu qing et al. (2022) analyzed the sports culture tourism resources in Beijing and Zhang, and drew the conclusion that the polarization effect of "point", the diffusion effect of "axis" and the driving effect of "surface" of the tourism area were expanded [4]. Lapointe Dominic et al. (2016) explore the spatial and location changes of coastal tourism destinations due to climate change adaptation, and identify strategies to combine tourism development and climate change adaptation to optimize space [5].

On the other hand, the spatial structure characteristics of tourist areas are affected by many factors [6]. The law of spatial heterogeneity points out that if an independent variable has an impact on a dependent variable among spatial entities, then the spatial distribution of the independent variable and the dependent variable should be consistent [7]. This theory provides a theoretical basis for verifying the influence of different influencing factors on the spatial distribution characteristics of tourism resources by methods such as geographic detectors. At present, geographical detectors have been widely used in the research of public health, regional economy and other aspects. For example, Ren Li min et al. (2023) used geographic detectors to study the influencing factors of the novel coronavirus [8], and Li Yin et al. (2024) used geographic detectors to study the spatio-temporal evolution and driving factors of land urbanization [9].In addition, in the study of factors affecting the spatial distribution of tourism resources, most scholars mainly use field investigation case analysis, and rarely use models.For example, when Zhang Yanyu et al. (2020) studied the factors affecting the spatial distribution characteristics of natural tourist attractions in Jiangxi Province, they concluded that natural factors and human factors affect the distribution of scenic spots [10].In studying the factors affecting the spatial distribution of high-level scenic spots in the Yellow River Basin, Li Donghua et al(2020) used buffer analysis tools, the number of scenic spots around the Yellow River, the number of traffic and other investigations to find that the spatial distribution of high-level scenic spots in the Yellow River Basin was affected by the natural geographical environment and traffic location [11].

In general, at present, experts and scholars have conducted in-depth research on the spatial distribution characteristics and influencing factors of tourism resources, and have made great progress. However, the current research on the spatial distribution characteristics and influencing factors of tourism resources lacks effective research models, and the research conclusions are not persuasive.

In view of the lack of effective research models on the spatial distribution characteristics and influencing factors of tourism resources, this paper uses geographical detectors to study the influencing factors and verifies the validity of the model.

According to the characteristics of big data processing, such as timeliness, fast speed and authenticity [12], this paper obtains the point of interest (POI) data of Shaoxing tourist attractions through Autonavi open platform API interface (https://lbs.amap.com/).

The main purpose of this paper is to study the spatial distribution characteristics and influencing factors of scenic spots in Shaoxing, an ancient historical and cultural city in China. In this paper, spatial statistical analysis techniques such as mean nearest neighbor analysis, nuclear density analysis, unbalance index analysis, and standard deviation ellipse are used to describe the distribution characteristics of scenic spots, such as spatial aggregation, distribution equilibrium, and spatial orientation, and geographical detectors are applied to the study of factors affecting the distribution characteristics of scenic spots. In order to verify the role of geographical detectors in the study of factors affecting the spatial distribution characteristics of scenic spots, so as to provide scientific basis for the planning, layout and development of tourist attractions in Shaoxing and its similar cities, and then promote the high-quality development of tourism in Shaoxing and its similar historical and cultural ancient cities.

## Materials and methods

### Research area

Shaoxing City is located in the central and western part of Zhejiang Province, between 29°13′36″ and 30°16′17″ north latitude, and between 119°53′02″ and 121°13′38″ east longitude, and is located in the junction of three major geomorphic units, namely, West Zhejiang Mountains and hills, East Zhejiang hills and North Zhejiang Plain. The terrain from the north Shaoyu plain to the south gradually transition to hilly mountains, high in the south and low in the north, the landform is complex. The city is rich in water resources, densely covered with rivers and numerous lakes. The city is 130.03 km long from east to west, 116.86 km wide from north to south, with a total area of 8,279 km^2^, under the jurisdiction of three districts, two cities and one county (Yuecheng District, Keqiao District, Shangyu District, Zhuji City, Shengzhou City, Xinchang County). In 2022, the city’s GDP is 735.055 billion yuan, the domestic tourism income is about 40.242 billion yuan, and the overseas tourism income is about 1.36 million US dollars. Shaoxing City is one of the first national historical and cultural cities [13].

### Data source and processing

**Fig 1:**
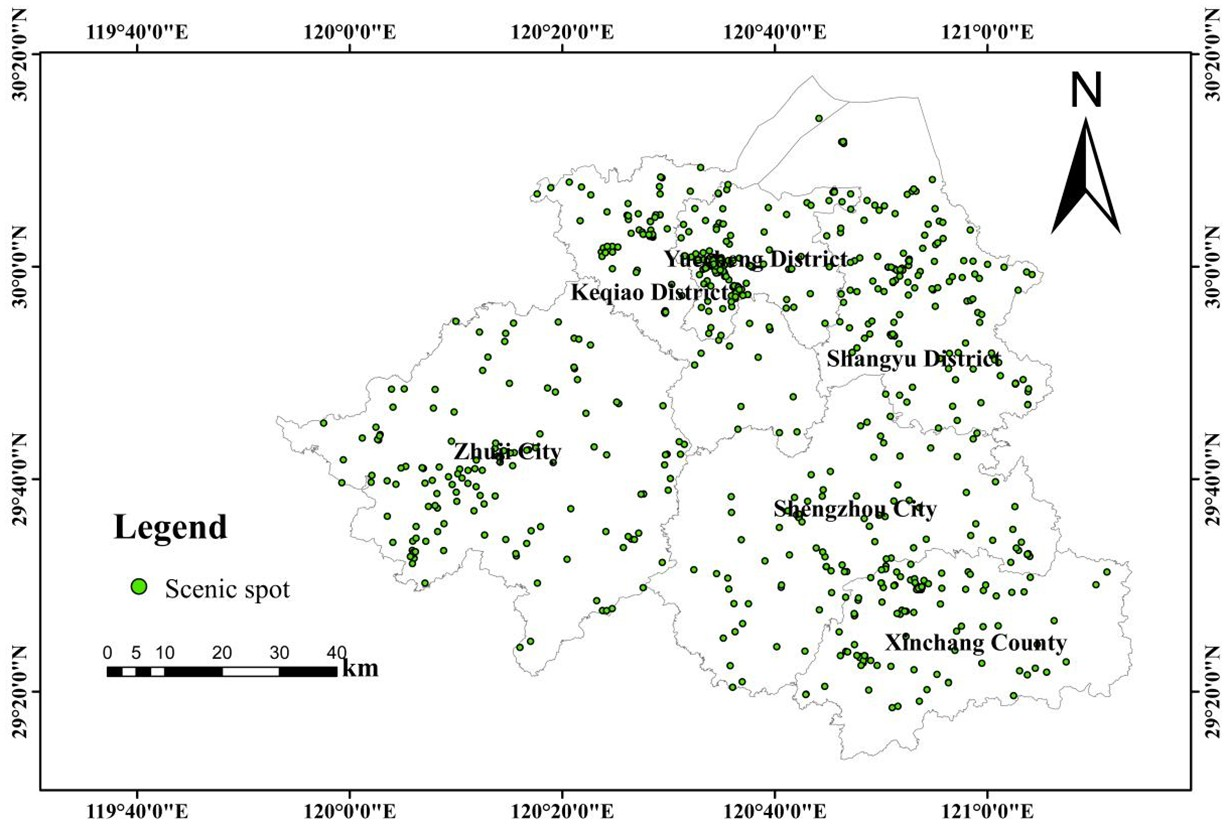
Spatial visualization distribution map of tourist attractions in Shaoxing.

In this paper, the data of points of interest (POI) come from Autonavi Open platform API. According to Autonavi POI classification and coding, 779 pieces of data of tourist attractions in Shaoxing are extracted by keyword search for "scenic spots" and "museums", etc. The data acquisition time is 01/2024, and a series of data processing operations such as data cleaning, de-duplication and merging are carried out. Finally, 726 scenic spot POI data were retained, including national scenic spots, provincial scenic spots, temples, temples, museums, memorials and other tourist attractions. The obtained POI data of scenic spots are shown in Table 1. The ArcGIS10.2 software was used to transform and project the POI scenic spot data in coordinate space, and realize the spatial visualization of the POI data of Shaoxing scenic spot (Fig 1).

**Table 1:** Examples of some tourist attractions in Shaoxing.

Name of scenic spot	region	longitude	dimensionality	category	address
Lanting Scenic area	Keqiao District	120.50	29.93	National scenic spot	Lanting Street Lanting Village
Cao E river	Shangyu District	120.89	30.02	park	119 Jiangdong Road
Shaoxing opera town	Shengzhou City	120.78	29.52	Tourist attraction	1 Wenyue Road, Shijia ’ao Village, Ganlin Town
Giant Buddhist temple	Xinchang county	120.89	29.50	National scenic spot	117 West Renmin Road
East Zhejiang Canal Museum	Yuecheng District	120.52	30.04	museum	1596 West Fenglin Road
Lu Xun’s hometown	Yuecheng District	120.58	30.00	Memorial hall	241 Lu Xun Middle Road
Shaoxing East Lake	Yuecheng District	120.63	30.00	National scenic spot	Cloud East Road, Gaobu Street
Xi Shi’s hometown	Zhuji City	120.24	29.70	Memorial hall	2 Zhuluo East Road
……					

#### Average nearest neighbor analysis

The average nearest neighbor analysis method can quantitatively describe the proximity degree and spatial distribution of geographical elements. The process is to first measure the distance between each particle and its neighbors, then calculate the average of these distances, and compare the calculated average with the expected average of the particles in the case of random distribution to calculate the nearest neighbor ratio [14].

The formula of average nearest neighbor analysis is as follows:

$$ANN={\bar{D}}_{O}/{\bar{D}}_{E}$$ (1)

$${\bar{D}}_{O}=\sum_{\text{i}=1}^{n}d_{\text{i}}/n$$ (2)

$${\bar{D}}_{E}=0.5/\sqrt{n/A}$$ (3)

Where: *ANN* is the average nearest neighbor ratio. ${\bar{D}}_{O}$ Is the average distance between tourist attractions in the region, *d*_*i*_ is the distance between scenic spot i and its nearest scenic spot, and the distance in this paper adopts Euclidean distance. *n* is the total number of attractions. ${\bar{D}}_{E}$ Is the expected mean value of scenic spots in the case of random distribution.A is the total area.It can be seen that if *ANN* < 1, the scenic spots are clustered; *ANN* = 1, the scenic spots are distributed with the model; *ANN* > 1, the scenic spots are distributed.

#### Kernel density analysis

Kernel density analysis can be used to describe the degree of spatial agglomeration of geographical elements [15], using ArcGIS software can calculate the density of spatial point elements in the surrounding environment, and intuitively represent the degree of aggregation of spatial points or lines. The larger the kernel density value, the denser the surface area elements are in space.

Kernel density analysis formula:

$$f(x)=\frac{1}{nh}\sum_{\text{i}=1}^{n}K(\frac{x-x_{\text{i}}}{h})$$ (4)

Where:*f*(x) is the kernel density function value at the space attraction *x*. *n* is the number of tourist attractions in the region. (*x*-*x*_*i*_) is the distance from scenic spot *x* to scenic spot *x*_*i*_. *h* is the bandwidth,that is, the search radius calculated by the kernel density. *K*(.) Is the kernel density function.

#### Unbalance index analysis

Unbalance index analysis can be used to describe the equilibrium degree of spatial distribution of geographical elements. In this paper, Lorentz curve is used to calculate the unbalance index [16].

Imbalance index formula:

$$S=\frac{\sum_{i=1}^{n}Y_{i}-50(n+1)}{100n-50(\text{n}+1)}$$ (5)

Where:*n* is the sum of the number of districts (counties and cities) under Shaoxing City,and *Y*_i_ is the *i* cumulative percentage after the proportion of the number of scenic spots under Shaoxing district (counties and cities) in Shaoxing city from large to small. The imbalance index ranges from 0 ≤ *S* ≤ 1. If *S* = 0 the scenic spots are evenly distributed in all districts of Shaoxing, that is, the scenic spots are evenly distributed; if *S* = 1,the scenic spots are concentrated in one area, and the distribution is extremely uneven.

#### Standard deviation ellipse

Standard deviation ellipse is one of the methods used to analyze the directionality of spatial point data. Using standard deviation ellipse, the centrality and directionality of data point distribution can be analyzed from a spatial perspective [17]. In this paper, the standard deviation ellipse contains 68% of the number of attractions [18]. Specifically, the standard deviation ellipse takes the arithmetic mean center of spatial point data as the center of the ellipse, and then calculates the standard deviation in the X and Y directions according to the mean center, so as to determine the length and length semi-axis and direction of the ellipse. Moreover, the long semi-axis of the ellipse represents the direction of the distribution of scenic spots, while the short semi-axis represents the range of the distribution of scenic spots. It shows that the direction of the scenic spot is more obvious.

Standard deviation ellipse calculation formula:

1. Center of ellipse:
$$SDE_{x}=\sqrt{\frac{\sum_{i=\text{1}}^{n}{\text{(}x_{i}-\bar{\text{X}}\text{)}}^{\text{2}}}{n}}\;SDE_{y}=\sqrt{\frac{\sum_{i=\text{1}}^{n}{\text{(}y_{i}-\bar{\text{Y}}\text{)}}^{\text{2}}}{n}}$$ (6)
2. Rotation Angle of ellipse:
$$\text{tan}\theta =\frac{\sum_{i=\text{1}}^{n}{{\tilde{x}}_{i}}^{2}-\sum_{i=\text{1}}^{n}{{\tilde{y}}_{i}}^{2}+\sqrt{{\left(\sum_{i=\text{1}}^{n}{{\tilde{x}}_{i}}^{2}-\sum_{i=\text{1}}^{n}{{\tilde{y}}_{i}}^{2}\right)}^{2}+4{\left(\sum_{i=\text{1}}^{n}{\tilde{x}}_{i}{\tilde{y}}_{i}\right)}^{2}}}{2\sum_{i=\text{1}}^{n}{\tilde{x}}_{i}{\tilde{y}}_{i}}$$ (7)
3. Axis of ellipse:
$$\sigma_{x}=\sqrt{2}\sqrt{\frac{\sum_{i=1}^{n}{\left({\tilde{x}}_{i}\;\text{cos}\;\theta -{\tilde{y}}_{i}\;\text{sin}\;\theta \right)}^{2}}{n}}\;\sigma_{y}=\sqrt{2}\sqrt{\frac{\sum_{i=1}^{n}{\left({\tilde{x}}_{i}\;\text{sin}\;\theta +{\tilde{y}}_{i}\;\text{cos}\;\theta \right)}^{2}}{n}}$$ (8)

Where: (*x*_*i*_, *y*_*i*_) is the spatial coordinates of scenic spots, ($\bar{\text{X}}$,$\bar{\text{Y}}$) is the average center coordinates of all scenic spots, and *n* is the number of scenic spots.*θ* is the elliptic direction Angle, taking the positive north as 0°, and the clockwise rotation to the major axis is *θ*. ${\tilde{x}}_{i}$ and ${\tilde{y}}_{i}$ are the difference between the arithmetic mean center *x*_*i*_ and *y*_*i*_.

#### Geographic detector

Geodetectors are a set of statistical methods for detecting spatial differentiation and revealing the driving forces of its differentiation, including four detectors (differentiation and factor detection, interaction detection, risk area detection, ecological detection). The basic idea is that if an independent variable has an important influence on a dependent variable, then the spatial distribution between the two variables should also have a certain similarity. In this paper, differentiation, factor detection and interaction detection are used to analyze the explanatory power of factors affecting the spatial distribution of scenic spots.

Differentiation and factor detection formula:

$$q=1-\frac{\sum_{h=1}^{L}N_{h}\sigma_{h}^{2}}{N\sigma^{2}}=1-\frac{SSW}{SST}$$ (9)

$$SSW=\sum_{h=1}^{L}N_{h}\sigma_{h}^{2},SST=N\sigma^{2}$$

Where:*h* is the layer of influence factorX,which is divided into L layers.*N*_*h*_ is the number of units in layer *h*, *N* is the number of units in the whole region, and N in this paper is the 6 districts (counties and cities) of Shaoxing City.$\sigma_{h}^{2}$ and *σ*^2^ are the variances of stratification *h* and whole region *Y*, respectively.0≤ q ≤1, the larger the value of *q* the more obvious the spatial differentiation of the dependent variable *Y*, that is, the stronger the explanatory power of the corresponding influence factor *X* to the dependent variable *Y*.

## Results and discussion

### Analysis of spatial aggregation state of scenic spots

Based on the POI data of scenic spots in Shaoxing City, the average nearest neighbor analysis method of ArcGIS10.2 software was used to calculate the spatial aggregation status of scenic spots in Shaoxing City and its subordinate districts (counties and cities) (Table 2). The analysis results show that:

1. The nearest neighbor ratio of the scenic spots in Shaoxing City and its subordinate districts (counties and cities) is less than 1, and the Z score is less than -2.58, and the P value is less than 0.01, which passes the significance test of 0.01 confidence level. It can be preliminically determined that the scenic spots in Shaoxing city and its subordinate districts (counties and cities) are clustered rather than randomly distributed.
2. In addition, the concentration degree of scenic spots in Yuecheng District and Shangyu District is higher, especially the nearest neighbor ratio of Shangyu district is 0.664, which is more obvious, while the concentration degree of scenic spots in Xinchang County, Keqiao District, Shengzhou and Zhuji city is relatively low. Preliminary analysis of the possible reasons are that Yuecheng District and Shangyu District are adjacent to each other, the cross-regional correlation of scenic spots is relatively large, and the economy of the two districts is relatively developed, the transportation is relatively convenient, and the government supports the tourism industry. The terrain of Shengzhou City and Zhuji city is more complex, and the traffic is backward compared with Shangyu District and Yuecheng District.

**Table 2:** Nearest neighbor ratio and spatial status distribution of districts (counties and cities) in Shaoxing.

Region name	Regional area (km^2^)	Mean observation distance (km)	Expected mean distance (km)	Nearest neighbor ratio	Z score	P value	state
Shaoxing City	8279	1.191	1.688	0.706	-15.180	0.000	cluster
Shangyu District	1406	1.068	1.608	0.664	-7.491	0.000	cluster
Yuecheng District	493	0.561	0.837	0.670	-8.364	0.000	cluster
Xinchang county	1214	1.154	1.676	0.689	-6.192	0.000	cluster
Keqiao District	1066	1.514	1.885	0.803	-3.2580	0.001	cluster
Shengzhou City	1789	1.829	2.229	0.820	-3.262	0.001	cluster
Zhuji City	2311	1.716	2.024	0.848	-3.463	0.001	cluster

Note: Regional area data are from Shaoxing Statistical Yearbook 2023.

### Analysis of spatial distribution pattern of scenic spots

**Fig 2:**
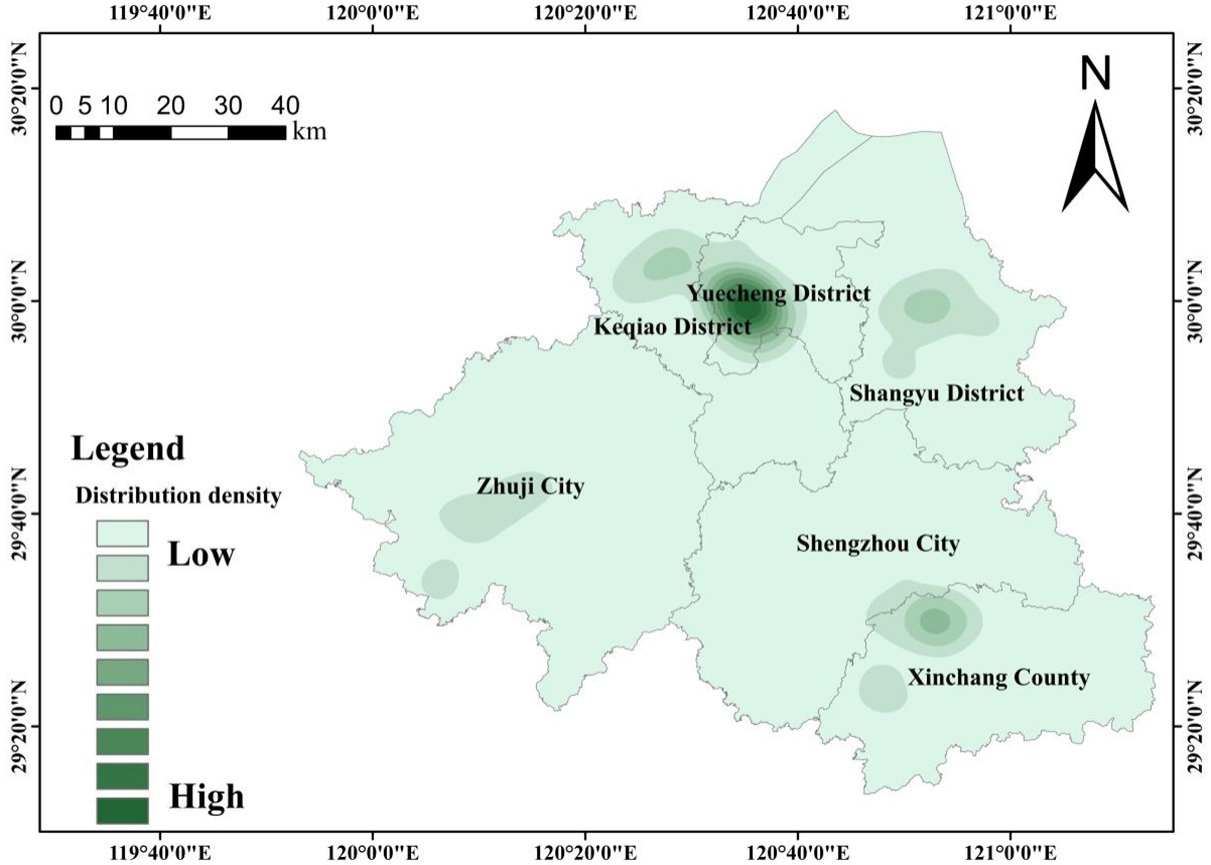
Kernel density map of spatial distribution of scenic spots in Shaoxing.

Based on the POI data of scenic spots in Shaoxing City, the kernel density analysis method of ArcGIS10.2 software was used to analyze the spatial distribution pattern of scenic spots in Shaoxing City, and the spatial distribution kernel density map of scenic spots in Shaoxing City was formed (Fig 2). The overall distribution of scenic spots is characterized by higher density in the north and lower density in the south. There are obvious differences in the distribution of scenic spots in different counties.

The specific analysis is as follows:

1. The city of Shaoxing presents a distribution pattern of "one center and more points, gradually spreading outward". "One center" refers to a group of natural and cultural scenic spots such as "Shu Sheng’s hometown", "Shaoxing Museum" and "Fushan Park" in Yuecheng District, the main city of Shaoxing, with "Lu Xun’s Hometown—Shen Garden" as the core;
2. The regional distribution pattern of Shaoxing City presents "one center, multiple points, gradually expanding outward". "One center" refers to the 5A-level scenic spot "Lu Xun’s Hometown—Shen Garden" in Yuecheng District, the main city of Shaoxing, which is surrounded by a number of natural and cultural attractions such as "Hometown of Calligraphy Saint", "Shaoxing Museum", "Fushan Park"; "Multiple points" refer to the Cao’e River Tourist Area in Shangyu District, which is mainly composed of "China Filial Piety Park", "Shangyu Museum", "Jiangshang Garden", the landscape and cultural tourist area in Keqiao District, which is mainly composed of "Jianhu Lake", "Keyan Scenic Area", "Keqiao Ancient Town", and the natural and cultural tourist area in Xinchang County, which is mainly composed of "Xinchang Buddha ", "Gushan Park".
3. Each district (county, city) in Shaoxing City basically has its own core tourist attractions and forms an inter-district linkage pattern. For example, at the boundaries of Keqiao District—Yuecheng District and Shengzhou City—Xinchang County, core attractions have driven the development of surrounding attractions, especially cross-district ones.

### Analysis of balanced distribution of scenic spots

According to the POI data of scenic spots in Shaoxing City, the number of scenic spots in each district (county, city) in Shaoxing city was summarized, and the percentage and cumulative percentage of the number of scenic spots in each district (county, city) were calculated according to the analysis method of imbalance index. The statistical results are shown in Table 3.

**Table 3:** Statistics of scenic spots in districts (county, city) in Shaoxing.

District (county, city)	Number of scenic spots	percent	Cumulative percentage
Yuecheng District	176	24.24%	24.24%
Zhuji City	141	19.42%	43.66%
Shangyu District	136	18.73%	62.39%
Xinchang county	108	14.88%	77.27%
Shengzhou City	90	12.40%	89.67%
Keqiao District	75	10.33%	100.00%

**Fig 3:**
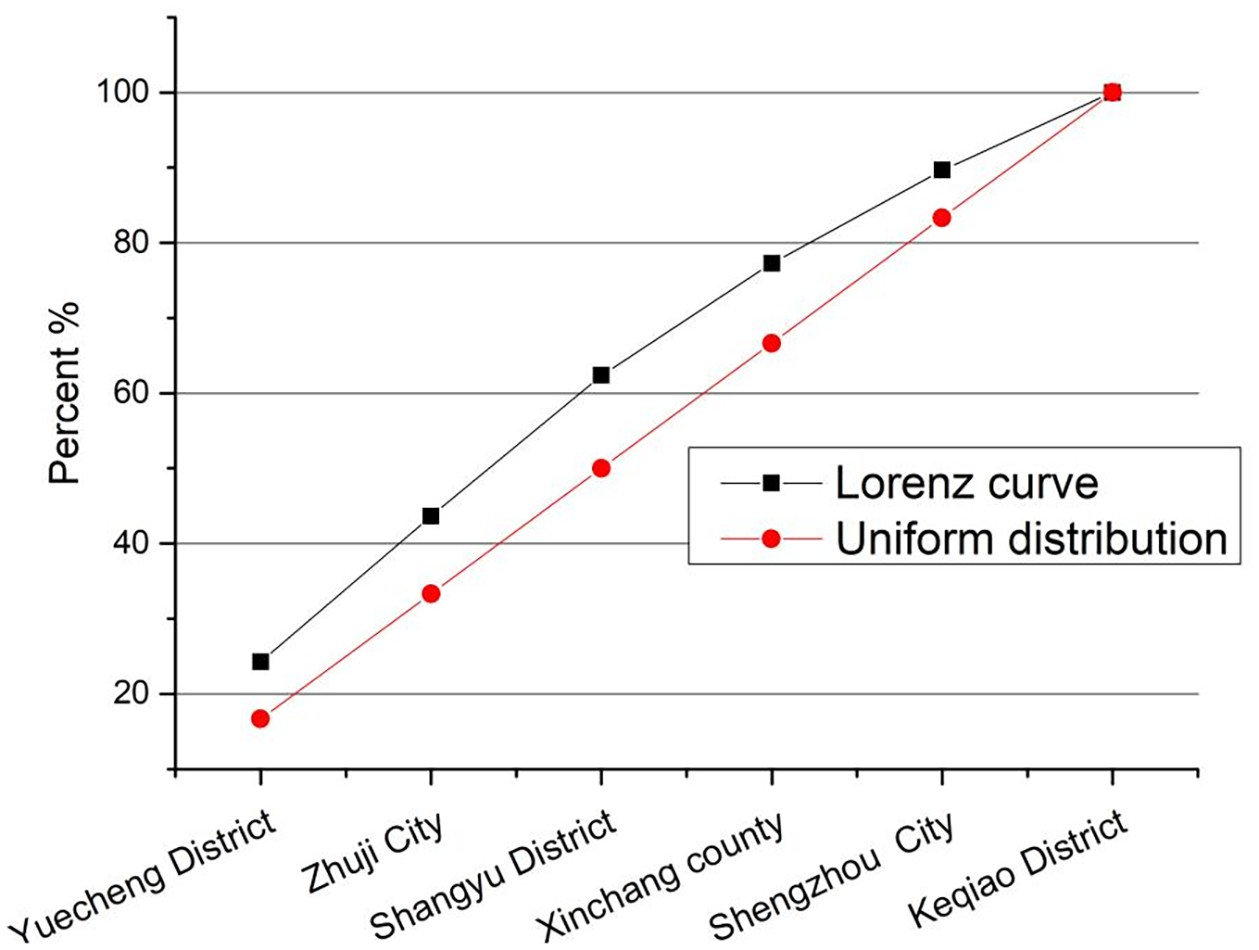
Lorenz curve of scenic spot distribution in Shaoxing.

As shown in Table 3, the number of scenic spots in Yuecheng district accounted for the highest proportion, reaching 24.2%, followed by Zhuji City with 19.42%. The number of scenic spots in Keqiao District accounts for the least, only 10.33%. According to the imbalance index calculation, the imbalance index of scenic spots in Shaoxing city = 0.1889, indicating that the number of scenic spots in Shaoxing city is relatively evenly distributed among all districts (counties and cities). According to the Lorentz curve of the spatial distribution of scenic spots in each district (county and city) of Shaoxing city (Fig 3), it can also be seen that the distribution number of scenic spots in each district (county and city) of Shaoxing city is not very different.

From the analysis of chapter 2.2 spatial distribution pattern of scenic spots, it can be seen that on the one hand, scenic spots in Shaoxing City show a pattern of "one center and multiple points, gradually spreading", and each district has its own core tourist attractions, such as Lu Xun’s hometown—Shen Garden "5A-level scenic spot in Yuecheng District. Shangyu District Caoejiang scenic zone, Keqiao District Keqiao Ancient town—Keyan Scenic area landscape and cultural scenic spots, so that the district (county, city) scenic spots, scenic spots development is relatively balanced; On the other hand, due to the linkage development of scenic spots in various districts (counties and cities), such as the development and construction of the Tang Poetry Road cultural belt in eastern Zhejiang and the Canal cultural belt in eastern Zhejiang, the development of scenic spots in various districts (counties and cities) is relatively balanced.

### Spatial directivity analysis of scenic spots

**Fig 4:**
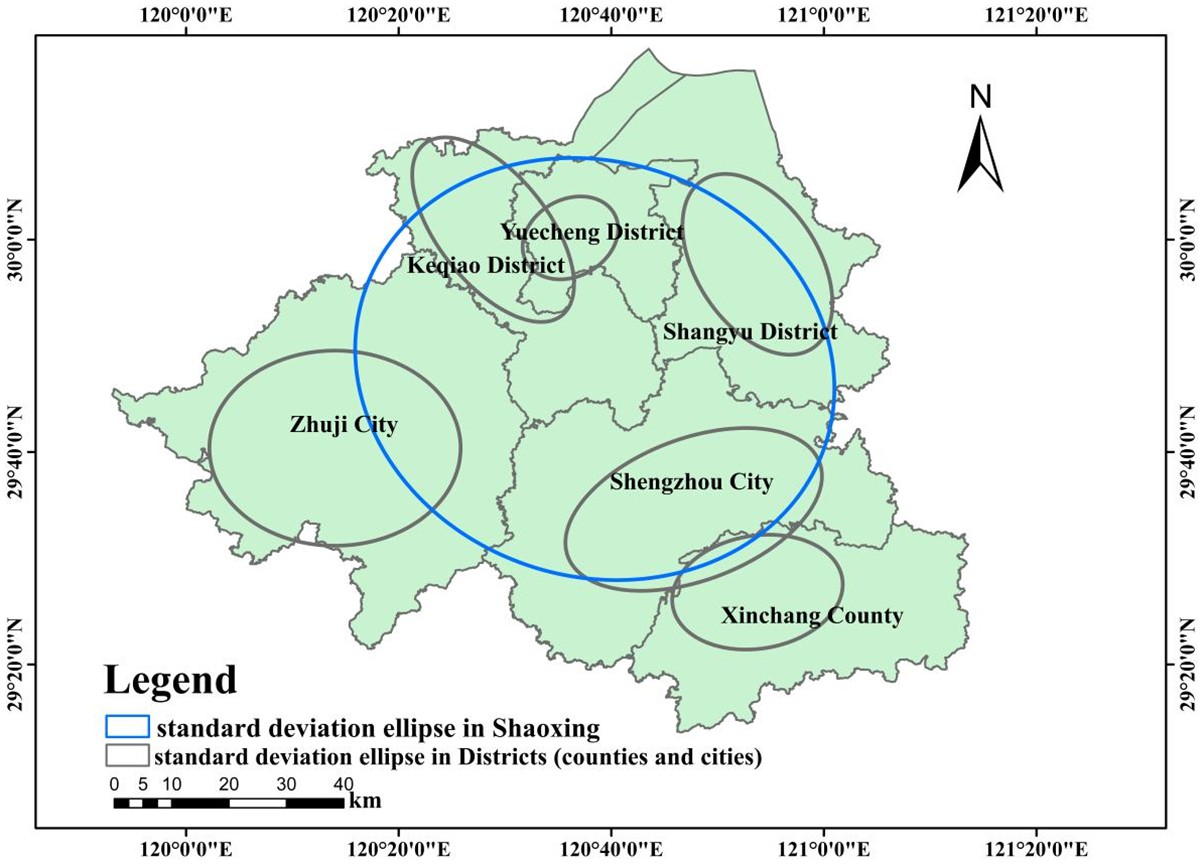
Standard deviation ellipse in districts (counties and cities) in Shaoxing.

Based on the POI data of scenic spots in Shaoxing City, the ArcGIS10.2 software was used to analyze the standard deviation ellipse (directional distribution) of the scenic spots in Shaoxing City and its subordinate districts (counties and cities), and the orientation and distribution trend of the scenic spots in Shaoxing City and its subordinate districts (counties and cities) were studied. The results were shown in the distribution diagram of standard deviation ellipse (Fig 4) and the parameter table (Table 4).

**Table 4:** Standard deviation ellipse parameters in districts (counties and cities) in Shaoxing.

Region name	area (km^2^)	X-axis length (km)	Y-axis length (km)	Angle (o)
Shaoxing City	4180.53	38.26	34.81	136.52
Yuecheng District	160.12	6.40	7.96	43.41
Keqiao District	497.46	18.25	8.68	145.67
Shangyu District	507.05	16.90	9.55	151.88
Zhuji City	1017.98	16.98	19.08	88.27
Shengzhou City	792.58	12.09	20.86	62.34
Xinchang county	404.36	9.79	13.15	74.60

From the result of standard deviation ellipse, it can be seen that the distribution direction of scenic spots in Shaoxing city is basically consistent with the direction of regional shape of Shaoxing city. The distribution direction of scenic spots in Shaoxing City is southeast to northwest and includes most areas except Xinchang County, indicating that the distribution of scenic spots in Shaoxing city is relatively uniform, which is in line with the conclusion of chapter 2.3 analysis of the balanced distribution of scenic spots.

Specifically, the distribution direction of scenic spots in Keqiao District, Shangyu District, Zhuji City and Shengzhou City is basically consistent with the shape of their respective regions, indicating that the distribution of scenic spots in the above regions is relatively uniform, while the scenic spots in Yuecheng District are mainly distributed in the southwest of the region, and the scenic spots in Xinchang County are mainly distributed in the northwest of the region, and the uniformity of the distribution of scenic spots is relatively poor.

The reason is that there is the only 5A-level scenic spot "Luxun’s hometown—Shenyuan Scenic Spot" in the southwest of Yuecheng District, while the 4A-level scenic spot Xinchang Temple is located in the northwest of Xinchang County, and the distribution of Yuecheng District and Xinchang County indicates that the concentration effect of scenic spots is more obvious.

From the difference of X axis and Y axis, the difference of Keqiao District is 9.57km, that of Shengzhou City is 8.77km, that of Shangyu District is 7.35km, that of Xinchang County is 3.36km, that of Zhuji City is 2.10km, and that of Yucheng District is 1.56km, indicating that the distribution of scenic spots in Keqiao District, Shengzhou City and Shangyu District has strong directionality. However, the directionality of Xinchang County, Zhuji City and Yuecheng District is relatively weak. The short axis length of Zhuji city and Shengzhou city is the largest, indicating that the distribution range of scenic spots in Zhuji city and Shengzhou City is larger.

From the Angle of elliptic deflection, the deflection Angle of Keqiao District and Shangyu District is the largest, and the deflection Angle is basically consistent with the direction of Shaoxing scenic spots, which is southeast to northwest direction. The deflection Angle of Yuecheng District, Zhuji City, Shengzhou City and Xinchang County is less than 90°, and the distribution direction of scenic spots is northeastern-southwest.

### Analysis of influencing factors of spatial distribution characteristics of scenic spots

#### Index acquisition and processing

According to the above chapter 2.1–2.4, the preliminary analysis of the clustering state, distribution pattern, equilibrium and orientation of scenic spots space shows that the spatial distribution of scenic spots is influenced and affected by many factors [19]. Based on this, a total of 9 indicators (Table 5) from the three dimensions of natural environment, social environment and economic environment are used as independent variables for detection, and the kernel density value of scenic spots in various districts (counties and cities) of Shaoxing is used as dependent variable to explore the influencing factors of the spatial distribution of scenic spots.

**Table 5:** Factors affecting the spatial distribution of tourist attractions.

dimensionality	index	data source
Natural environment	(X_1_)elevation	Resources and Environmental Science and Data Center, Chinese Academy of Sciences
	(X_2_)Vegetation coverage	Shaoxing Natural Resources and planning Bureau
	(X_3_)Water system area proportion	Shaoxing Natural Resources and planning Bureau
Social environment	(X_4_)Government policy	Government work report
	(X_5_)Permanent population	Shaoxing Statistical Yearbook
	(X_6_)Proportion of highway mileage open to traffic	Shaoxing Statistical Yearbook
Economic environment	(X_7_)Reception capacity	Shaoxing Statistical Yearbook
	(X_8_)Tourism income	Shaoxing Statistical Yearbook
	(X_9_)Gross regional product (GDP)	Shaoxing Statistical Yearbook

The following are some explanations for each index: (X_1_) Elevation data refers to the average altitude of scenic spots. The elevation data can be obtained by downloading DEM from the website of Resources and Environmental Science and Data Center of Chinese Academy of Sciences and using the ArcGIS10.2 software to obtain the average altitude of all scenic spots; The vegetation coverage area was obtained from Shaoxing Natural Resources and Planning Bureau, and the data included the total area of forest land, wetland and grassland. (X_2_) The vegetation coverage rate was equal to the ratio of the vegetation coverage area to the area of the district (county or city); The water system area comes from Shaoxing Natural Resources and Planning Bureau, and the data includes the sum of the area of river water surface, lake water surface, reservoir water surface, pit water surface, ditches, etc. (X_3_) The proportion of the water system area is equal to the ratio of the water system area to the area of the district (county, city). (X_4_) The sum of the frequency of tourism and scenic spots mentioned in the annual government work report of each district (county and city) of Shaoxing; (X_5_) resident population, (X_6_) highway mileage, (X_7_) reception capacity, (X_8_) tourism revenue, and (X_9_) gross regional product are obtained from the 2023 Shaoxing Statistical Yearbook, where (X_6_) highway mileage is equal to the ratio of highway mileage to the area of the district (county or city). (X_7_) Reception capacity is the sum of the number of star-rated hotels in each district (county, city).

#### Result analysis of influencing factors

The differences, factors and interactions of the above 9 indicators were detected by geographical detector, and the influence of each index factor on the spatial distribution of scenic spots was analyzed.

#### Differentiation and factor detection

q values of differentiation and factor detection results are shown in Table 6.

**Table 6:** Spatial distribution differentiation of scenic spots and factor detection results.

index	X_1_	X_2_	X_3_	X_4_	X_5_	X_6_	X_7_	X_8_	X_9_
q value	0.4430	0.4495	0.4430	0.3211	0.1734	0.2504	0.9877	0.3741	0.4430

1. *Natural environmental factors*. Among the natural environmental factors, the elevation q value of (X_1_) is 0.4430, the vegetation coverage q value of (X_2_) is 0.4495, and the water system area proportion q value of (X_3_) is 0.4495. It can be seen that the explanatory power of X_1_, X_2_ and X_3_ on the spatial distribution of scenic spots is not much different, and they are all relatively important. On the one hand, a certain altitude, vegetation coverage and water system area are the initial and necessary factors for the formation of tourist attractions; On the other hand, within a certain altitude, a unique natural landscape is often formed, reflecting the type of scenic spots, such as temples, waterfalls and other scenic spots often exist at a higher altitude, while the former residences of celebrities, museums and so on are generally at a lower altitude. Parks and other scenic spots tend to have higher vegetation coverage. Areas with more developed river and lake water systems tend to have more human activities, and Shaoxing, as a famous water town city, has more developed water systems, and the area ratio of water systems is also more important. Therefore, the altitude, vegetation coverage rate and water system area ratio have relatively high explanatory power.
2. *Social environmental factors*. The explanatory power of social and environmental factors to the spatial distribution of scenic spots in descending order is (X_4_) government policy q value 0.3211, (X_6_) highway mileage ratio q value 0.2504, (X_5_) resident population q value 0.1734. Among them, government policies have the greatest explanatory power. For example, in the 14th Five-Year Plan for Culture and Tourism of Shaoxing City, it is mentioned that Yuecheng District should dig deep resources of "Jishan Water", build Dongjianhu national wetland Park, and improve the quality and expand Kuaiji Mountain Tourism Resort. Keqiao District should be guided by large projects and built into "scenic city, scenic town and scenic village" to promote the innovation and development of all-region tourism; Xinchang County should rely on the resources and conditions of unique natural mountains and rivers, history and culture, food specialties and other resources to implement the construction of cultural elegance, Tianmu Mountain development, the construction of Tang poetry cultural city, the construction of nineteen Peaks tourist attraction, and the revitalization of rural scenic spots. It can be seen that policy factors play a positive role in guiding and adjusting resources in tourism and the layout of scenic spots and scenic spots, and the role is crucial. In addition, the greater the proportion of highway traffic mileage, the higher the accessibility of the scenic spot, the stronger the ability of the scenic spot to attract tourists, the more permanent population, the greater the number of visits to the scenic spot, to a certain extent, promote the spatial aggregation of scenic spots.
3. *Economic and environmental factors*. Among the economic and environmental factors, (X_7_) reception capacity q value of 0.9877 has the greatest explanatory power to the distribution pattern of scenic spots, (X_9_) gross regional product q value of 0.4430 is second, and (X_8_) tourism income q value of 0.3741 is relatively low. Where attractions gather, the reception capacity is higher, and the reception capacity is higher, which in turn promotes the aggregation of attractions, so the aggregation of attractions and reception capacity form a mutually promoting role. The gross regional product often reflects the overall situation of the local economy. In areas with good economic development, transportation and other infrastructure are often developed, which can promote the development of tourism and the development of local tourist attractions, thus affecting the distribution and development of scenic spots. Tourism income can reflect the level of regional tourism development to a certain extent, and the increase of tourism income can promote the development of local tourism, thus promoting the distribution and aggregation of tourist attractions.

#### Interaction detection

The interaction of geographical detectors can be used to detect the interaction of various influencing factors on the spatial distribution of scenic spots. The interaction detection results are shown in Table 7. It can be seen from Table 5 that the explanatory power of the 9 index factors after interaction is significantly enhanced than that of the single factor, and the results are manifested as two-factor enhancement and nonlinear enhancement. It shows that the spatial distribution characteristics of scenic spots are influenced by many factors. In particular, the ratio of (X_4_) government policy to (X_1_) elevation, (X_2_) vegetation coverage, (X_3_) water system area, (X_6_) highway mileage, (X_5_) resident population to (X_1_) elevation, (X_2_) vegetation coverage and (X_3_) water system area, When (X_9_) GDP interacts with (X_6_) highway mileage and (X_8_) tourism income, the explanatory power increases to 1; when (X_7_) reception capacity interacts with (X_4_) government policies and (X_5_) resident population, the explanatory power increases to 0.9999; when (X_9_) GDP interacts with (X_4_) government policies and (X_5_) resident population, the explanatory power increases to 0.9999. It shows that the interaction of these factors plays the most important role in the spatial distribution of scenic spots in Shaoxing city.

**Table 7:** Detection results of spatial distribution interaction of scenic spots.

	**X**_**1**_	**X**_**2**_	**X**_**3**_	**X**_**4**_	**X**_**5**_	**X**_**6**_	**X**_**7**_	**X**_**8**_	**X**_**9**_
**X**_**1**_	0.4430								
**X**_**2**_	0.5423	0.4495							
**X**_**3**_	0.5358	0.5423	0.4430						
**X**_**4**_	1.0000	1.0000	1.0000	0.3211					
**X**_**5**_	1.0000	1.0000	1.0000	0.9999	0.1734				
**X**_**6**_	0.9965	0.9955	0.9965	1.0000	0.4939	0.2504			
**X**_**7**_	0.9900	0.9955	0.9900	0.9999	0.9999	0.9955	0.9877		
**X**_**8**_	0.9965	0.9965	0.9965	0.9995	0.4820	0.9965	0.9965	0.3741	
**X**_**9**_	0.5359	0.5458	0.5359	0.9999	0.9999	1.0000	0.9901	1.0000	0.4430

## Conclusions

This paper obtains the data of points of interest (POI) in Shaoxing City from Autonavi Open Data Platform as the research object, and adopts the ArcGIS10.2 software and spatial statistical analysis methods such as average nearest neighbor analysis, nuclear density analysis, imbalance index analysis and geographic detector to conduct statistical analysis on the spatial pattern of scenic spots in Shaoxing City and its sub-districts (counties and cities). The spatial distribution characteristics and influencing factors of tourist attractions in Shaoxing City were studied. The specific conclusions are as follows: In terms of distribution characteristics, the scenic spots of Shaoxing city and its subordinate districts (counties and cities) are clustered rather than randomly distributed, and the degree of clustering of scenic spots in Yuecheng District and Shangyu District is relatively high. The overall distribution of scenic spots in Shaoxing City is characterized by high density in the north and low density in the south. Regional scenic spots show a distribution pattern of "one center point, gradually spreading outward", and the number of scenic spots is relatively evenly distributed among the districts (counties and cities), and the number of distribution is not different. The distribution direction of scenic spots in the city and its subordinate districts (counties and cities) is basically consistent with the direction of their respective regions, and the distribution of scenic spots in their respective regions is also relatively uniform. In terms of influencing factors, natural environmental factors, social environmental factors, economic environmental factors have a certain impact on the distribution of scenic spots. The natural environment is the basis and basic condition of the distribution of scenic spots, the social environment and the economic environment promote the distribution of scenic spots, and the explanatory power of the two-factor interaction is obviously stronger than that of the single factor. This result is similar to the conclusion that the spatial distribution of scenic spots is affected by natural environmental factors, social environmental factors and economic environmental factors. For example, Guo Yanping et al. (2021) concluded through investigation that the distribution of scenic spots in Shanxi is affected by natural and social factors [20]. In this study, the explanatory power of the single factor is confirmed by the geographical detector, and the explanatory power of the Gemini influence is stronger than that of the single factor.

This study has a positive and far-reaching impact on the layout and optimization of scenic spots in Shaoxing City and other similar historical and cultural ancient cities. First, Shaoxing city should increase the development and optimization of the layout of scenic spots in its subordinate districts (counties and cities) as a whole, that is, optimize the distribution pattern of scenic spots in the whole area, so as to enable the linkage development of scenic spots in counties and cities. For example, increase the development and construction of the eastern Zhejiang Tang Poetry Road cultural belt and the eastern Zhejiang Canal cultural belt. Secondly, all districts (counties and cities) should fully tap their regional advantages and develop tourism according to local conditions. For example, Xinchang County and Zhuji City are rich in natural environment and resources, which can increase the construction of natural scenic spots and carry out ecological and healthy leisure Tours. Yue Cheng District has deep cultural deposits, can open up cultural tourism routes and build historical and cultural cities. In addition, natural resources are the basis for the development of tourism, and the protection of natural resources should be increased. At the same time, it is necessary to promote the economic development of various regions, so as to improve the reception capacity of scenic spots and the accessibility of scenic spots.

Finally, the paper still has some shortcomings, such as the subjectivity in data cleaning. Secondly, the obtained POI data of scenic spots include natural scenic spots, humanistic scenic spots, etc., and there is a lack of classified research on scenic spots. In the follow-up study, the data sources will be expanded and the types of scenic spots will be precise, so as to explore the influencing factors among various scenic spots and the interaction among scenic spots, in order to promote the positive development of Shaoxing and its similar historical and cultural ancient cities.

## Supporting information

S1 Data Datasets used in the research. (XLS)
